# In silico molecular and functional characterization of a dual function antimicrobial peptide, hepcidin (GIFT-Hep), isolated from genetically improved farmed tilapia (GIFT, *Oreochromis niloticus*)

**DOI:** 10.1186/s43141-023-00579-6

**Published:** 2023-11-21

**Authors:** K. L. Dhanya Lenin, Swapna P. Antony

**Affiliations:** Department of Marine Biology, Microbiology and Biochemistry, School of Marine Sciences, Cochin University of Science and Technology, Fine Arts Avenue, Kochi, Kerala 682016 India

**Keywords:** HAMP1 and 2, GIFT-Hep, Iron regulation, Antimicrobial peptide, Aquaculture, Genetically improved farmed tilapia, Therapeutic

## Abstract

**Background:**

Antimicrobial peptides (AMPs), innate immune response molecules in organisms, are also known for their dual functionality, exemplified by hepcidin—an immunomodulator and iron regulator. Identifying and studying various AMPs from fish species can provide valuable insights into the immune profiles of aquaculturally significant fish, which can be made use of in its culture.

**Results:**

Hepcidin, a dual-function antimicrobial peptide, was isolated from the gill tissue of Genetically Improved Farmed Tilapia (GIFT-Hep). GIFT-Hep consists of a 90 amino acid pre-propeptide with a 24-mer signal, a 40-mer propeptide, and a 26-mer mature peptide region. The mature peptide had a molecular weight of 3015.61 Da, a theoretical pI of 8.78, a net charge of +4.25, and a protein-binding potential of 2.06 kcal/mol. Four disulfide bonds were formed by eight cysteine residues in the mature region. The presence of positively charged arginine residues renders the peptide 50% hydrophobic. Molecular analysis of GIFT-Hep revealed the presence of a furin propeptide convertase motif, RX(K/R)R, which facilitates trimming of the peptide to yield the mature GIFT-Hep. The hypothetical iron regulatory sequence, QSHLSL, was also identified in the mature peptide. *In silico* predictions about the characteristics of GIFT-Hep, such as charge, hydrophobicity, high surface accessibility, transmembrane helical regions, hydrophobic faces, hot spots, and cell-penetrating properties, suggest that the peptide functions as an iron regulatory antimicrobial agent.

**Conclusions:**

This study reports a hepcidin antimicrobial peptide with both HAMP1 and HAMP2 properties isolated from genetically improved farmed tilapia, and further evaluation of the properties will prove the feasibility of GIFT-Hep being used as a therapeutant in aquaculture.

## Background

Fish, cold-blooded aquatic scaly vertebrates, are of interest to humans owing to their role as a global food resource. Aquaculture, which is governed by the physical, chemical, and biological factors of the environment, requires precise management to ensure sustainable production. The rapid advancements in the aquaculture sector have beseeched the need for research and breakthrough knowledge in the management of fish resources. Disease, a significant constraint, hampers the seamless operation of the sector, necessitating new approaches in treatment due to the emergence of antibiotic resistance. The solution to this challenge lies in the effective utilization of antimicrobial peptides (AMPs), which are natural host defense molecules. AMPs constitute an innate immune response with a broad spectrum of activity against bacterial, viral, fungal, and parasitic pathogens. Their specific mode of action reduces the risk of developing resistance.

Hepcidins constitute a class of short, cysteine-rich AMPs earlier referred to as LEAP-1 and 2 (liver-expressed antimicrobial peptide). They were initially discovered in human blood ultrafiltrate [1, 2] and simultaneously in human urine [3]. The name “hepcidin” indicates its high synthesis and expression rate in the liver (hep-) with significant microbicidal activity (-cidin). Shortly after its identification, hepcidin was also linked to iron metabolism in mice [4, 5]. The presence of basic amino acids, which impart a positive charge to the peptide, coupled with its amphipathic secondary structure, endows it with antimicrobial properties. Additionally, hepcidin serves other roles such as mediating cytokine-mediated inflammatory responses and addressing respiratory infections [6].

To date, hepcidin has been identified in various species including hybrid striped bass [7], bony fish [8], zebrafish [9], red sea bream (*Chrysophrys major*) [10], *Paralichthys olivaceus* [11], Nile tilapia (*Oreochromis niloticus*) [12], black porgy (*Acanthopagrus schlegelii*) [13], Antarctic notothenioid fishes [14], black rockfish (*Sebastes schlegelii*) [15], gilthead seabream [16], olive flounder (*Pseudosciaena crocea*) [17], orange-spotted grouper (*Epinephelus coioides*) [18], turbot (*Scophthalmus maximus* L.) [19], *Brachymystax lenok* [20], Mandarin fish (*Siniperca chuatsi*) [21], three tilapia species [22], and Nile tilapia [23].

The Genetically Improved Farmed Tilapia (GIFT), an aquaculture-relevant strain of *Oreochromis niloticus*, has gained significant momentum in India. It offers a rapid and cost-effective source of animal protein to the population, thereby bolstering the aquaculture sector in recent years. Research on strategies to address diseases, antibiotic resistance, and food security is crucial for the advancement of GIFT culture. The present study focuses on identifying AMPs from GIFT that can contribute to maintaining healthy and biosafe aquaculture practices.

## Methods

### Experimental organism and ethical committee approval

The experimental organism, Genetically Improved Farmed Tilapia (GIFT— *Oreochromis niloticus*), was obtained from the Rajiv Gandhi Centre for Aquaculture (RGCA), located in Vallarpadam, Kochi, India. The handling of experimental organisms and experiments conducted with them was approved by the Institutional Animal Ethical Committee.

### Tissue extraction, RNA isolation, and cDNA synthesis

One adult fish (31 cm in length and weighing 725 g) was selected for the study to facilitate targeted tissue extraction. The collected fish was transported to the laboratory in live condition and was immersed in an anesthetic bath with clove oil (0.2 ml in 500 ml water). The anesthetized fish was subjected to tissue extraction under sterile RNase-free conditions, and the extracted tissue was preserved in TRI reagent (Sigma) at −80 °C. RNA isolation was carried out following the manufacturer’s protocol. The quality and quantity of the isolated RNA were confirmed by gel electrophoresis and spectrophotometry. The gill RNA samples with an absorbance ratio (A_260_:A_280_) in the range of 1.8–1.9 were used for cDNA synthesis via RT-PCR. The standard reverse transcription procedure involved using 5 μg total RNA, 100 U of M-MuLV reverse transcriptase, 1× reverse transcriptase buffer, 2 mM dNTPs, oligo d(T)20, 3.5 mM MgCl_2_, and 20 U RNase inhibitor. The thermal cycle program included an initial RNA denaturation step (65 °C for 5 min), followed by the cDNA synthesis step (42 °C for 1 h), and, finally, the enzyme inactivation step (85 °C for 15 min) resulting in a cDNA yield from 5 μg of total RNA.

### PCR screening and sequencing of the gene of interest

The cDNA obtained was screened for the presence of hepcidin transcripts using gene-specific primers [24]. The PCR reaction was conducted in a 10 μl reaction volume with EmeraldAmp® PCR Master Mix (Takara Biomedical Inc., Japan) following the manufacturer’s protocol. The thermal cycle involved denaturation at 94 °C, annealing at 60 °C, and elongation at 68 °C. The resulting amplicons were subjected to 1.0 % agarose gel electrophoresis and visualized using the UV gel doc system (G:BOX, Syngene, UK). The PCR products were purified by Exo-CIP (New England Biolabs) treatment and subsequently sequenced on an ABI Prism 377 DNA sequencer at the AgriGenome Sequencing Facility in Kochi, India.

### In silico characterization of the gene of interest

The nucleotide sequence obtained was subjected to *in silico* characterization. The basic local alignment search tool (BLAST) available on the National Center for Biotechnology Information portal (https://blast.ncbi.nlm.nih.gov/) was utilized to identify significant alignments. The identity of the nucleotide sequence obtained as part of the present investigation was deciphered thereby. The translate tool ExPASy was used to deduce the amino acid sequence from the obtained nucleotide sequence. SignalP 5.0 tool (http://www.cbs.dtu.dk/services/SignalP/) was employed to detect the presence of a signal peptide, ProP 1.0 server (http://www.cbs.dtu.dk/services/ProP/) was used to check for the possible presence of a propeptide, and DeepLoc 1.0 (http://www.cbs.dtu.dk/services/DeepLoc-1.0/) was used to predict the protein subcellular localization. For further analysis, the APD3 antimicrobial peptide database (https://aps.unmc.edu/) and the ProtParam tool of ExPASy (https://web.expasy.org/protparam/) were utilized to decipher the physical, chemical, and antimicrobial properties of the peptide. Peptidase cleavage sites within the peptide were assessed using the online PeptideCutter tool (https://web.expasy.org/peptide_cutter/), while hydrophobic regions within the peptide were identified using the Kyte and Doolittle scale of the ProtScale tool (https://web.expasy.org/protscale/).

The aggregation propensity of the peptide was assessed using the AGGRESCAN 2d online server (http://bioinf.uab.es/aggrescan) [25]. The server identifies the short specific sequence stretches based on an aggregation-propensity scale derived from *in vivo* experiments involving natural amino acids. To identify active regions in AMPs, an *in silico* antimicrobial sequence scanning system called AMPA (http://tcoffee.crg.cat/apps/ampa/do) [26] was employed. Predictions regarding the physicochemical properties of the peptide in an intestine-like environment were made using the HLP web server (http://crdd.osdd.net/raghava/hlp/index.html) [27]. The helical arrangement and hydrophobic face of the peptide chain were identified by Heliquest web server (https://heliquest.ipmc.cnrs.fr/). Multiple sequence alignment of the peptide was done against similar sequences from other vertebrates using MEGA v.7 [28], and the phylogenetic tree was constructed by the neighbor-joining method. For deciphering the secondary structure, transmembrane topology, and biological process predictions, the PSIPRED 4.0 Protein Analysis Workbench by UCL-CS Bioinformatics (http://bioinf.cs.ucl.ac.uk/psipred/) was employed. The PEP-FOLD server (https://bioserv.rpbs.univ-paris-diderot.fr/services/PEP-FOLD/) was used for structure prediction [29, 30]. Protein structure prediction was conducted using Phyre2, the Protein Homology/Analogy Recognition Engine V 2.0 (http://www.sbg.bio.ic.ac.uk/phyre2), an automated protein structure homology-modeling platform. Additionally, 3D modeling was carried out using the SWISS-MODEL server (https://swissmodel.expasy.org/) an automated protein structure homology-modeling platform of the ExPASy server which was used for 3D modeling. The PROCHECK program (http://services.mbi.ucla.edu/PROCHECK/) [31] was employed to check the stereochemical quality of the peptide structure by analyzing the residue by residue and overall structure geometry using the Ramachandran plot [32]. The MolProbity score of the peptide was also noted. Furthermore, the peptide under investigation underwent analysis using AntiCP (https://webs.iiitd.edu.in/raghava/anticp/index.html), an online server utilizing the support vector machine (SVM) algorithm to predict anticancer peptides.

## Results

**Fig. 1:**
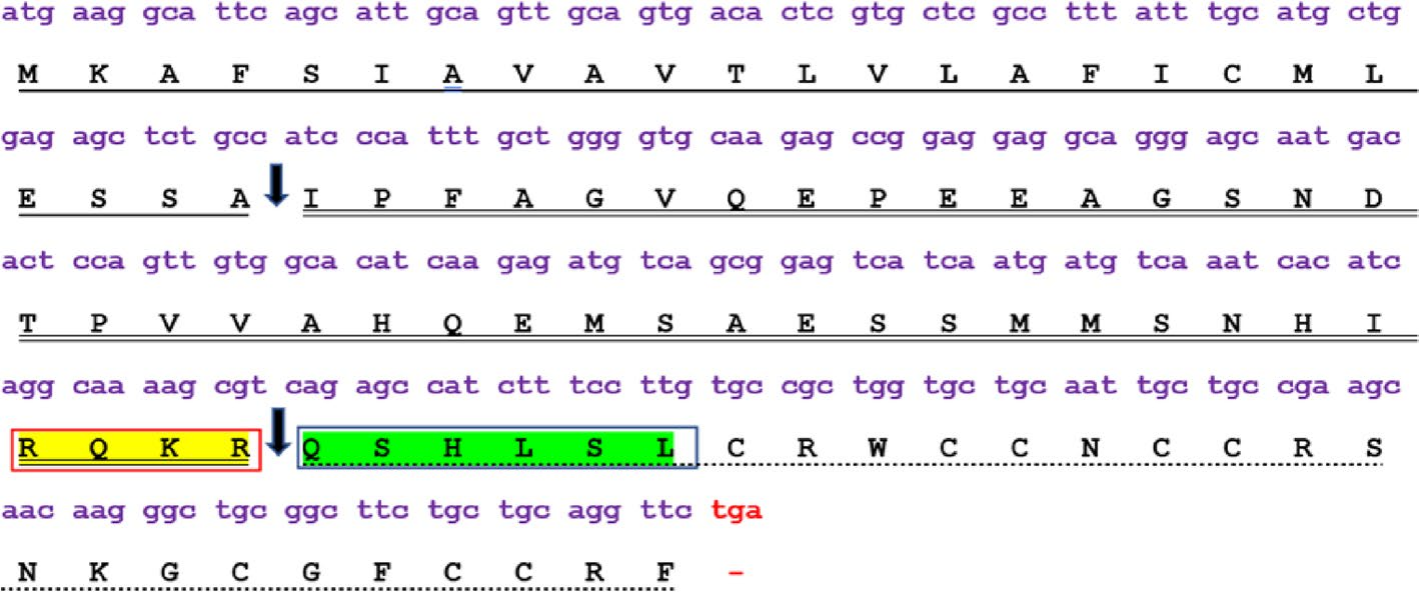
The open reading frame of 273 nucleotides encoding a 90 amino acid pre-propeptide, including a signal peptide (underline), a propeptide (double underline), and a mature peptide region of 26 amino acids (dotted underline). The red fond indicates the stop codon. The two filled black downward arrows indicate the signal peptidase cleavage site and the mature peptide cleavage site, respectively. The yellow highlight is the furin propeptide convertase motif, and the hypothetical iron regulatory sequence (QSHLSL) at the start of the mature peptide is green highlighted

A 273 bp nucleotide sequence was identified from the gill tissues of GIFT. Upon BLASTn analysis, the sequence was confirmed to be hepcidin, showing 100 % alignment to hepcidin-2 mRNA sequences of *Sarotherodon galilaeus* (MH651366.1) and *Oreochromis niloticus* (MH651359.1). Additionally, it displayed 99.27 % similarity to the hepcidin-2 mRNA of *Coptodon zillii* (MH651361.1). The ExPASy translational tool deduced a 90 aa peptide sequence. Subsequent BLASTp analysis revealed significant alignment with hepcidin-2 from *Coptodon zillii* (98.89 %, QB055763.1) and *Chelon ramada* (91.11 %, QBO59824.1), hepcidin from *Larimichthys crocea* (90.00 %, ABC18307.1), and hepcidin-1 from *Micropterus dolomieu* (87.78 %, ACD13025.1). Thus, the peptide identified was named GIFT-Hep (GenBank submission: MZ422437) and will be addressed so hereafter (Fig. 1).

SignalP 5.0 predicted an N-terminal 24-mer signal peptide, with a signal peptidase cleavage site between SSA^24^-I^25^, with a probability of 0.8705. The ProP 1.0 server predicted a 40-mer propeptide with a propeptide furin-specific cleavage site at position 64 (RQKR^64^-Q^65^SHLSL) (Fig. 1). The furin propeptide convertase motif R X (K/R) R [33] aids in this cleavage process, resulting in a mature peptide of 26 residues.

The mature peptide exhibited a molecular weight of 3015.61 Da and a theoretical isoelectric point (pI) of 8.78. The peptide is composed primarily of polar amino acids, with Cys (C) accounting for 31 % of the composition, followed by Ser (S) and Arg (R) at 12 %. In terms of stability, the peptide was projected to have a half-life of 0.8 h in mammalian reticulocytes *in vitro*, 10 min in yeast, and 10 h in *Escherichia coli in vivo*. The computed instability index, which stands at 70.18, categorizes the protein as unstable. The grand average of hydropathicity (GRAVY) was computed to be −0.077, and its aliphatic index was determined as 30. APD3 predicted an overall net charge of +4.25, accompanied by a hydrophobic ratio of 50 %. The Wimley-White whole-residue hydrophobicity of the peptide was calculated as −1.7, and the protein-binding potential (Boman index) was 2.06 kcal/mol. Upon sequence alignment of GIFT-Hep in APD3, 88.5 % similarity was noted with brown trout (*Salmo trutta*) hepcidin-1 [34] which showed anti-gram positive, anti-gram negative, and antifungal activity.

The AMPA scanning system analyzed the entire 90 aa peptide and identified a bactericidal stretch from S^57^ to R^89^, covering the mature peptide. A shorter stretch of 14 amino acids M^54^MSNHIRQKRQSHL^68^ was predicted to be a cell-penetrating peptide (CPP), which coincided with the N-terminal bactericidal region predicted by AMPA. The HLP web server indicated slightly higher surface accessibility for the CPP region, which pretty well explains its possible mode of action. Additionally, the HLP web server predicted a prolonged half-life for the peptide. GIFT-Hep was predicted to be of soluble type and localized extracellularly by the eukaryotic protein subcellular predictor. According to the Kyte and Doolittle scale, a hydrophobic stretch was anticipated at the amino-terminal end of the peptide, extending until residue G^29^. A comparatively shorter hydrophobic stretch was predicted from L^70^–C^74^ in the mature region. The AGGRESCAN online server identified three hot spot areas in the peptide. The first one covered the entire signal peptide region (M^1^–I^25^), with two additional short stretches observed in the N-terminal (L^68^–N^76^) and C-terminal (G^85^–F^90^) regions of the mature peptide.

**Fig. 2:**
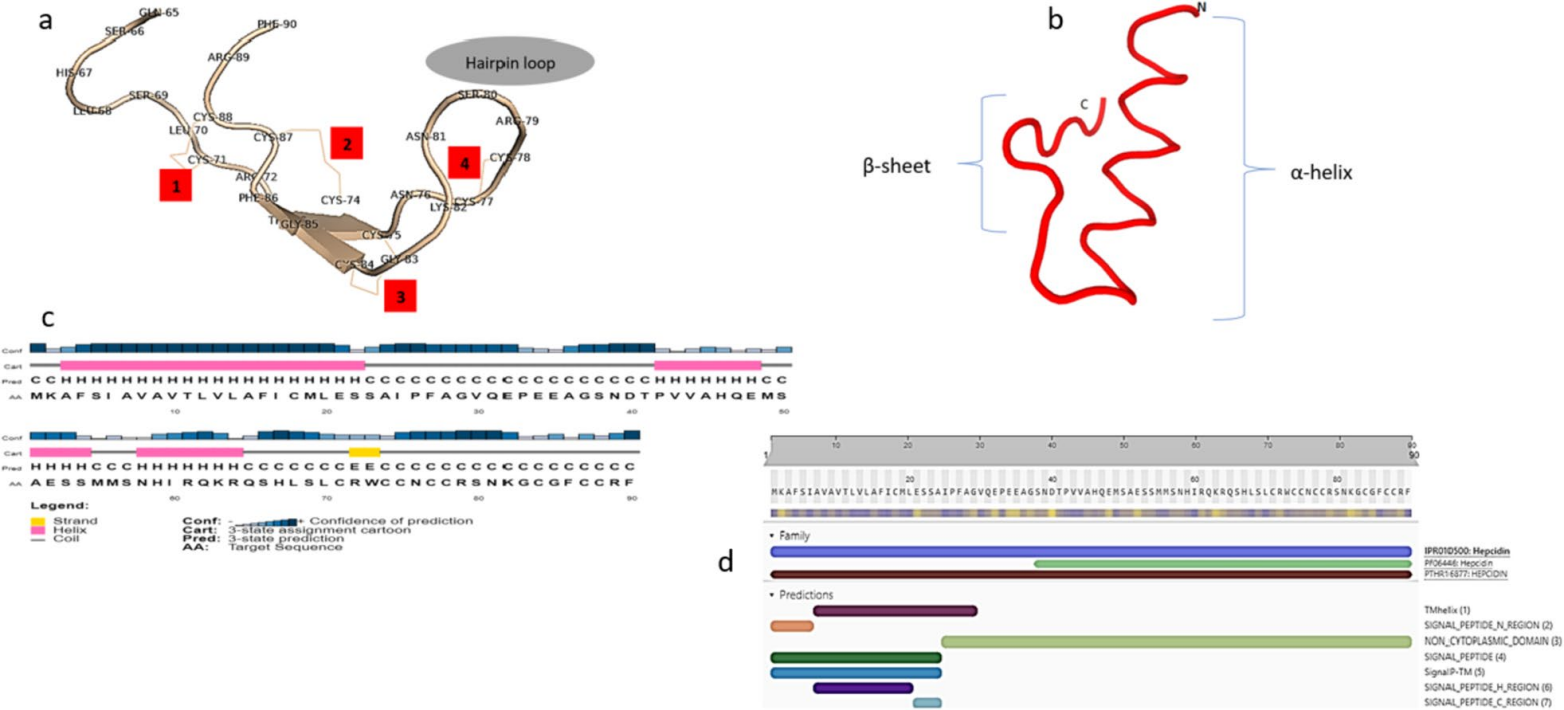
Structure prediction of the precursor GIFT-Hep. **a** The PyMOL predicted structure of the mature region showing antiparallel β-sheets with the characteristic disulfide bonds (C^71^–C^88^, C^74^–C^87^, C^75^–C^84^, and C^77^–C^78^) connecting the eight cysteines. The four S–S bonds are numbered, with the fourth one being a vicinal bond located in the hairpin loop. **b** The Mobyle predicted secondary structure displays the α-helical N-terminal signal peptide region, followed by a random propeptide region and the C-terminal β-sheet region. **c** The PSIPRED chart illustrates the helix, strand, and coiled regions in the peptide. **d** The amino acid types comprising the precursor GIFT-Hep, as predicted by the PSIPRED workbench. **e** The InterPro domain scan server predicted the peptide’s characteristics, identifying it as belonging to the hepcidin family in InterPro, Pfam, and Panther databases. A transmembrane helical region, non-cytoplasmic domain, and the signal peptide helix and coil region were also identified

**Fig. 3:**
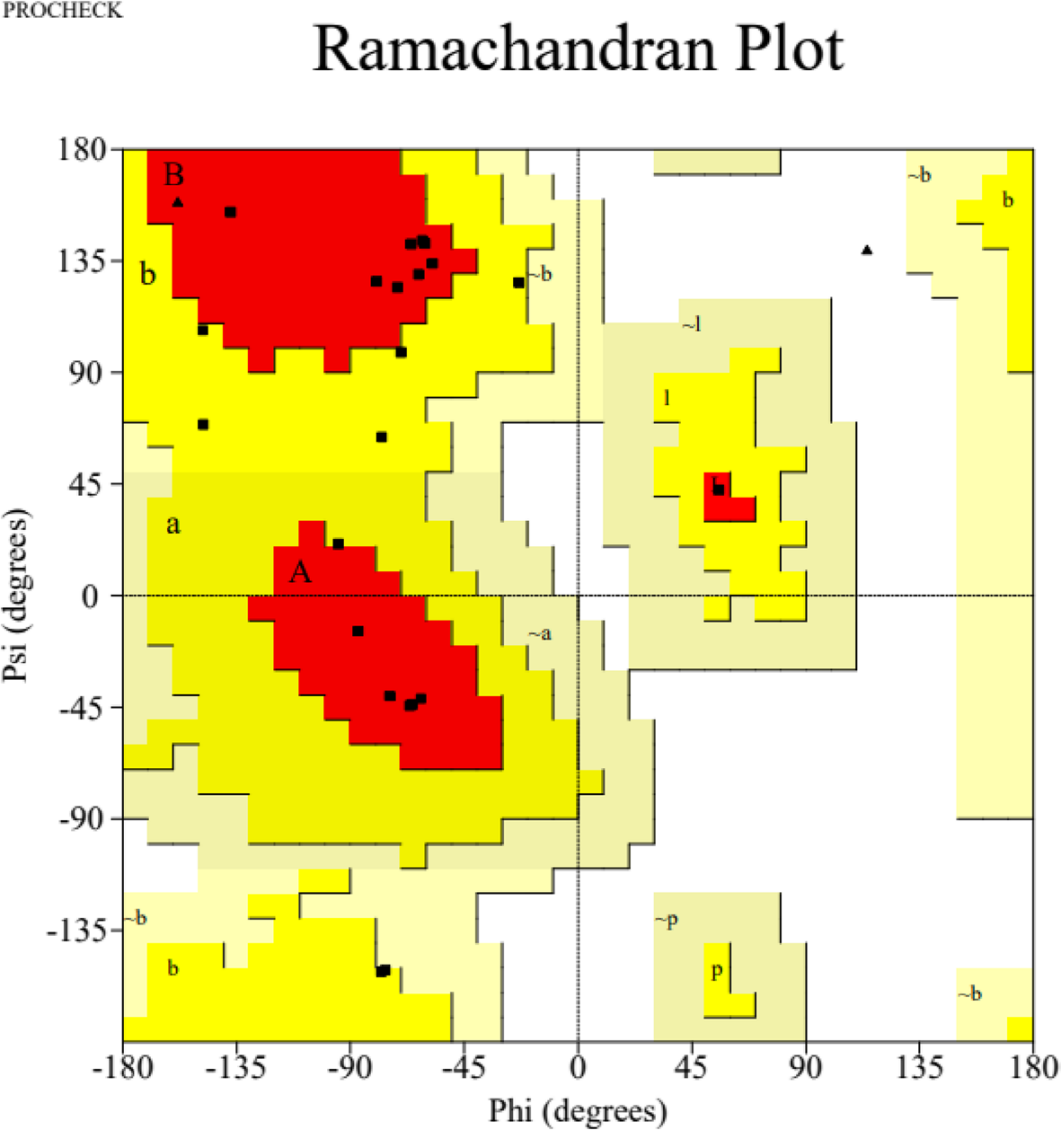
The Ramachandran plot of the GIFT-Hep illustrates the distribution of torsion angles, phi (φ), and psi(ψ) within its structure. The most favored regions A, B, and L (highlighted in red) contain 63.6 % residues, while the additional allowed regions a, b, l, and p (highlighted in yellow) contain 36.4 % residues. There are no residues reported in the generously allowed regions (~a, ~b, ~l, and ~p) or the disallowed regions. Upon analysis, a significant majority of the angles fall within the allowed regions of the plot, indicating a high-quality three-dimensional structure

**Fig. 4:**
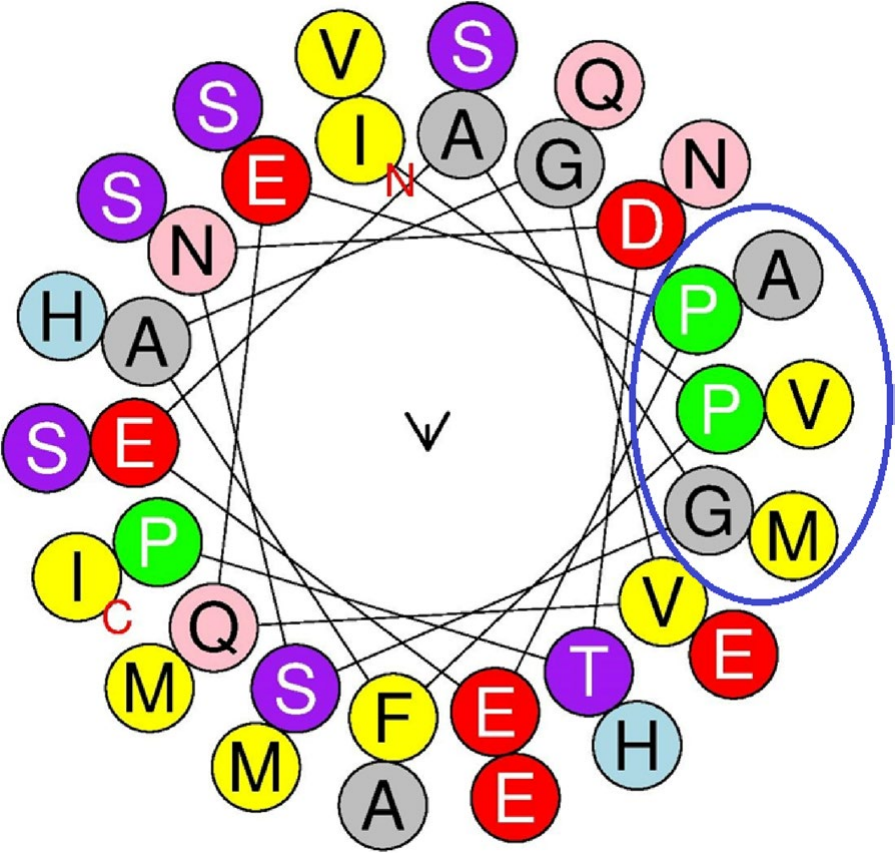
The helical wheel representation of GIFT-Hep. The Heliquest online tool was utilized to visualize the three-dimensional structure of the α-helical GIFT-Hep as a two-dimensional helical wheel projection. The hydrophobic face formed by the amino acids PAPVGM is highlighted within a circle, and the N- and C-terminals are indicated

The PDB file of the three-dimensional structure, deciphered by the SWISS-MODEL, was analyzed using PyMOL software to mark the disulfide bonds that stabilize the structure. The GIFT-Hep model was constructed using the template 1m4f.1.A on the SWISS-MODEL server. The predicted three-dimensional structural model displayed two anti-parallel beta-sheets in the mature region. Notably, disulfide bonding was identified between C^1^–C^8^, C^2^–C^7^, C^3^–C^6^, and C^4^–C^5^ (Fig. 2a). The Phyre^2^ server predicted a structure based on template c1s6wA, featuring a 58 % disordered region and a 65 % alpha-helical region. Additionally, the PEP-FOLD server predicted a structure with an α-helical N-terminal followed by a random helix and a C-terminal β-sheet structure (Fig. 2b). The PSIPRED workbench predicted a secondary structure with intermittent helix and coiled regions with a very short strand towards the C-terminal (Fig. 2 c). SWISS-MODEL predicted a MolProbity score of 1.79. The Ramachandran plot (Fig. 3) showed 63.6 % of residues in the favored region and 36.4 % (Arg^89^, Ser^69^, Lys^82^) as Ramachandran outliers. Furthermore, the helical wheel, as predicted by the Heliquest online tool, revealed a hydrophobic face formed by P-A-P-V-G-M amino acid residues (Fig. 4).

The MEMSAT tool predicted an initial six extracellular residues, followed by a transmembrane helical region involved in membrane interaction, spanning from A^7^ until S^22^. FFPred indicated significant probabilities for several biological processes, including the regulation of metabolic processes (GO:0019222), transport (GO:0006810), and cell surface receptor signaling pathway (GO:0007166). Molecular functions such as kinase activity (GO:0016301), cytokine activity (GO:0005125), growth factor activity (GO:0008083), and the cellular components’ prediction membrane (GO:0016020) and extracellular region (GO:0005576) were predicted with high probability. The InterPro Domain Scan predicted the cellular components as extracellular region (GO:0005576) and biological process of cellular iron ion homeostasis (GO:0006879). Additionally, the server attributed the investigated peptide to the hepcidin family within the InterPro, Pfam, and Panther databases (Fig. 2d).

**Fig. 5:**
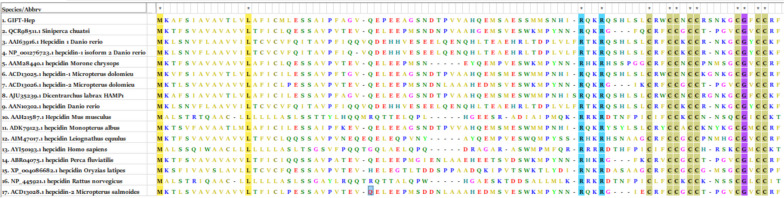
The ClustalW multiple sequence alignment of hepcidin sequences from fish and mammals conducted using MEGA7.0 software. The alignment highlighted the conserved eight cysteine residues along with the N-terminal methionine (M), leucine (L), arginine (R), and glycine (G) residues. The residues that are conserved at a 100% level are marked in black

**Fig. 6:**
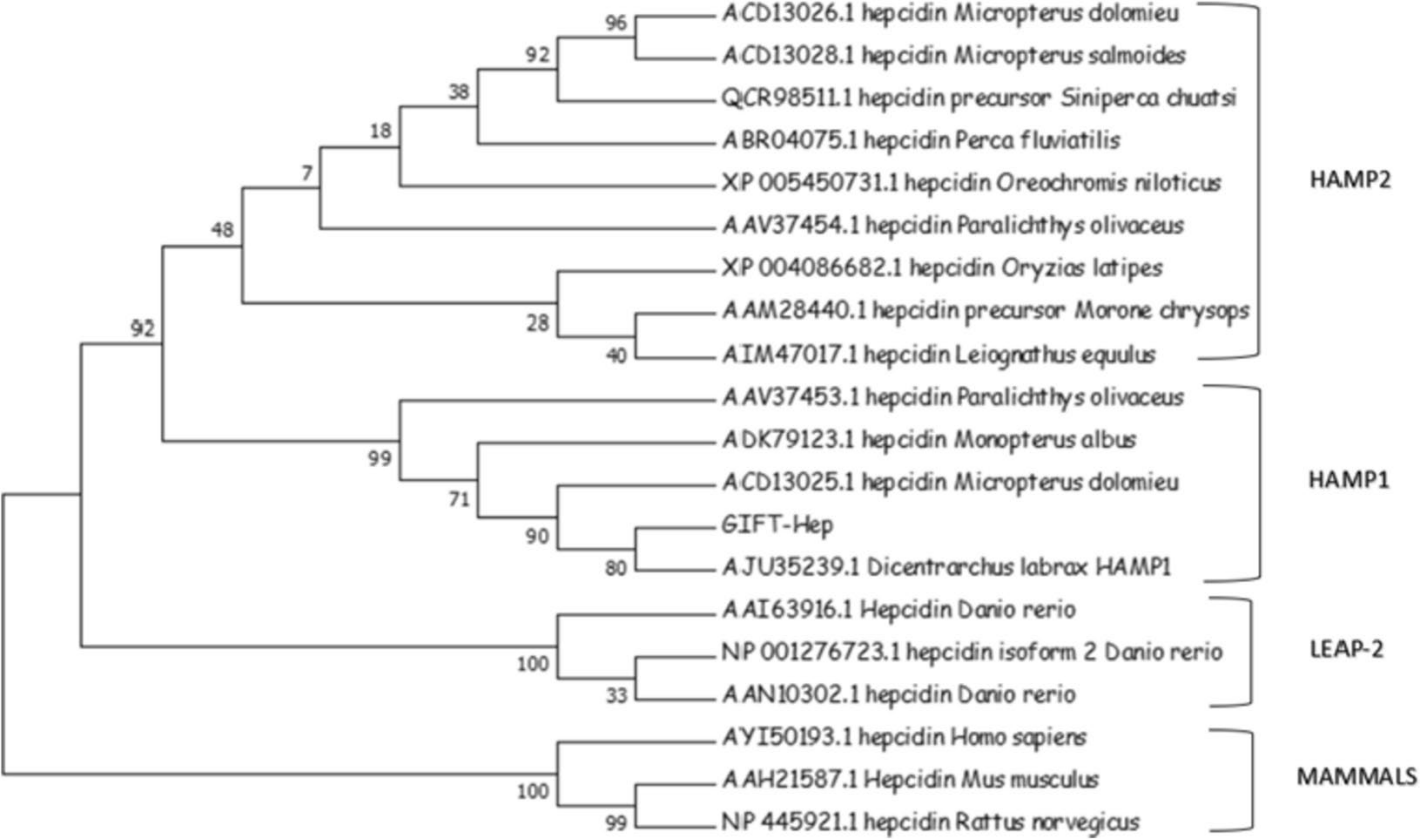
The phylogenetic tree constructed using MEGA 7.0 through the neighbor-joining method, based on hepcidin sequences from fishes and mammals. The hepcidin sequences from humans and rodents (mammals) are grouped in the outgroup, along with the hepcidin variants (LEAP-2) from *Danio rerio* forming a separate cluster. The HAMP 1 and HAMP 2 sequences are organized into distinct clades, with GIFT-Hep grouping alongside the HAMP1 sequence from *Dicentrarchus labrax*

The ClustalW multiple sequence alignment of the hepcidin sequences from various fishes and mammals revealed conserved cysteine residues within the mature region (Fig. 5). The neighbor-joining phylogenetic tree, constructed by the MEGA 7.0 software, exhibited distinct clusters. Mammalian hepcidins and LEAP-2 from *Danio rerio* formed separate outgroups, while the primary cluster consisted of fish hepcidins. Moreover, the fish hepcidin cluster was further divided into subclusters for hepcidin 1 and hepcidin 2 (Fig. 6).

## Discussion

Hepcidin serves diverse roles and can be broadly classified into HAMP1, regulating ferroportin linked to iron metabolism, and HAMP2, with antimicrobial functions [35]. The presence of a hypothetical iron regulatory sequence, Q-S-H-L-S-L [36], within GIFT-Hep categorizes it as HAMP1. In addition to hepatocytes which are considered the main source of hepcidin production, macrophages and adipocytes have also been found to express hepcidin mRNA at a lower scale [37]. These peptides are secreted as a prepropeptide, with an antimicrobial pre-(signal) region [34], followed by a propeptide and a mature region. While the signal peptide is conserved in numerous species, variations exist in the pro and mature regions. The hepcidin gene in teleost fish is comprised of three exons and two introns and is highly expressed in acidophilic granules [16]. In contrast to mammals, teleost fish possess 5–7 copies of hepcidin genes, a feature attributed to their diverse environmental habitats, necessitating greater quantitative and functional demands for the peptide [14].

Hepcidin levels were observed to be upregulated in the case of bacterial infection in Atlantic salmon [8] and striped sea bass [7]. The hep1 from *Scophthalmus maximus* L. was found to be expressed after microbial infection but was non-responsive to iron overload [19]. Whereas in sea bass, hepcidin was found to be upregulated both in case of iron overload and infection [38] and was found to be expressed in various tissues and played an overlapping role as an iron regulatory hormone as well as an antimicrobial agent [5, 23, 39]. Hepcidin in bass demonstrated antimicrobial activity against *Streptococcus iniae* (Gram-positive) and *Aeromonas salmonicida* (gram-negative), in addition to its activity against *E. coli* [40].

Hepcidin was found to exhibit high expression in the liver of gilthead seabream, Japanese flounder, Nile tilapia, sea bass, Mandarin fish, and black porgy. Additionally, it was also found to be expressed in tissues other than the liver which points to the fact that the expression profile differs species- and tissue-wise. When comparing the expression of HAMP1 and HAMP2 in sea bass, HAMP1 showed higher expression than HAMP2. Both were most abundantly expressed in the liver and leukocytes, followed by the pyloric caeca, stomach, mid-intestine, gill, and brain. Expression levels were notably low in the spleen, head kidney, trunk kidney, and anterior intestine [36].

The α-helix is an important secondary structure stabilized by hydrogen bonds between the C=O and N–H groups in the peptide backbone. α-helical peptides can be depicted through two-dimensional projections known as helical wheels [41]. GIFT-Hep adopts an α-helical conformation, and its helical wheel projections unveiled a hydrophobic face in its secondary structure conformation which hints at a possible microbial membrane interaction. Hydrophobic interactions, disulfide bridges, ionic bonds, and hydrogen bonds together make the protein structure stable, the breaking down of which leads to denaturation.

The folding and three-dimensional structural conformation of hepcidin is crucial for its DNA binding and subsequent antimicrobial activity [42]. The anti-parallel β-sheet along with a hairpin loop is stabilized by the disulfide bonds, while hydrogen bonds between the β-sheets confer amphipathicity. This characteristic is significant in many antibacterial and antifungal peptides, where the stable hairpin loop aids in binding to receptors like ferroportin, regulating iron homeostasis [43]. Although the presence of cysteine residues in the mature region is a common feature, their number and the resulting disulfide bonds formed can vary significantly. The notothenioid fishes were found to possess two disulfide bonds involving only four cysteine residues and exhibited greater structural stability [14]. Out of the four disulfide bonds formed in the case of GIFT-Hep (C1–C8, C2–C7, C3–C6, and C4–C5), one (C4–C5) was found to be a vicinal bond which has been attributed a functional significance [43]. The vicinal bond is found in the loop region of the peptide which has been identified as the most flexible part with high sequence diversity among species. This has led to the assumption that the loop region is one of the most functionally significant regions [40]. The 3D structure of GIFT-Hep also exhibits two antiparallel β-sheets with a hairpin loop and with four disulfide bonds, including one vicinal bond, which can be inferred to impart antimicrobial activity to the peptide in addition to it being an iron regulatory HAMP 1 peptide.

A Ramachandran plot visualizes the quality of torsional angles within a three-dimensional polypeptide chain structure. The two torsional angles, ψ (psi) and φ (phi) (Ramachandran angles), in the polypeptide chain refer to the backbone flexibility and the rotation it undergoes around these angles. For a stable structure, these angles are expected to be in the favored (allowed) region. GIFT-Hep has 63.6 % residues in the most favored region and 36.4 % residues in the additional allowed region.

Hepcidin acts either by disrupting the cell membrane organization [44] or by forming pores on the membrane. These pores function as channels, allowing the mature peptide to enter the cell and thereby inhibiting growth [45]. The FFPred Gene Ontology (GO) domains explicate functional characterization to proteins. On analysis, GIFT-Hep is found to have membrane association and antimicrobial activity. MolProbity is a structure-validation web service that provides an indication of the crystallographic resolution for the peptide [46]. A score of 1.79 for GIFT-Hep suggests a normal structure for the peptide.

The propensity for aggregation was found to depend on physicochemical properties such as hydrophobicity and secondary structure. The binding affinity of hot spots aids in protein-protein interactions [47], which can pose significant challenges in protein production, storage, and the recombinant production of bioactive peptides [25]. Identifying these active stretches can help prevent unwanted protein deposition and aid in designing more soluble variants of biotechnological importance [25, 48]. The hot spot area in the signal region of GIFT-Hep potentially contributes to its antimicrobial action by attaching to the bacterial membrane.

The hypothetical iron regulatory sequence Q(S/I)H(L/I)(S/A)L [49], which helps in binding to ferroportin, leading to its internalization and degradation and subsequently preventing the transport of iron across the membrane [50], was also identified in the N-terminal of the mature GIFT-Hep. Iron is essential for the growth and survival of microorganisms, and hepcidin with its role in iron metabolism will affect the growth of microorganisms, thereby acting as an antimicrobial agent as well. The presence of the iron regulatory sequence, along with the identified hot spots, attributes both iron regulatory and antimicrobial roles to the peptide. Given that iron is crucial for actively growing cells, introducing limitations in its availability could potentially emerge as a new technique in treating cancer [51]. The role played by hepcidin in iron metabolism is also highlighted in the coronavirus research scenario [52]. Also, the AntiCP server predicted anticancer properties of GIFT-Hep can be attributed to the presence of iron metabolism regulatory sequence in its mature region.

Tilapia hepcidin (TH)2–3 was used as a dietary supplement in grouper aquaculture. It induced multiple functions like increased superoxide dismutase activity, increased gut microbiota diversity, and also a differential regulation in the case of *Vibrio alginolyticus* infection all of which together contribute to the overall improvement in immunity of fishes [53]. The suboptimal *in vivo* half-life is a constrain in the therapeutic research of potential peptides [54], and the *in silico* analysis of GIFT-Hep predicted a feasible half-life for the peptide which is a positive attribute of its effectiveness as a candidate in therapeutic research.

Despite the promising future of AMPs in general, a few characteristics, such as broad-spectrum activity, instability, sensitivity to salt, hemolytic activity, and high production costs, serve as deterrents to their widespread application [55]. These aspects need to be addressed to ensure the effective utilization of AMPs as pharmaceuticals.

## Conclusions

The present study introduces a novel dual-function hepcidin from genetically improved farmed tilapia (GIFT-Hep). GIFT-Hep can be classified within the HAMP1 group due to the presence of the hypothetical iron-regulating sequence. However, *in silico* characterization has also associated it with HAMP2, which possesses antimicrobial properties. Further research is warranted to unravel the functional attributes and therapeutic potential of the peptide. These insights could be effectively harnessed for future applications in GIFT aquaculture. Discovering the therapeutic potential and strategies for implementing peptides from a species of aquacultural significance will undoubtedly stimulate its cultivation, thus contributing to economic and nutritional security within society.

## Data Availability

The datasets generated during and/or analyzed during the current study are available from the corresponding author on reasonable request.

## Abbreviations

GIFT-Hep: Genetically improved farmed tilapia-hepcidin
AMPs: Antimicrobial peptides
LEAP1: Liver-expressed antimicrobial peptide 1
HAMP1: Hepcidin antimicrobial peptide 1
HAMP2: Hepcidin antimicrobial peptide 2
RT-PCR: Reverse transcription polymerase chain reaction
